# A small molecule II-6s inhibits *Enterococcus faecalis* biofilms

**DOI:** 10.1080/20002297.2021.1978756

**Published:** 2021-09-14

**Authors:** Xinyi Kuang, Jin Zhang, Xian Peng, Qian Xie, Jiyao Li, Xuedong Zhou, Youfu Luo, Xin Xu

**Affiliations:** a State Key Laboratory of Oral Diseases & National Clinical Research Center for Oral Diseases, West China Hospital of Stomatology, Sichuan University, Chengdu, China; b Department of Cariology and Endodontics, West China Hospital of Stomatology, Sichuan University, Chengdu, China; c Department of Endodontics, College of Dentistry, University of Illinois at Chicago, Chicago, Chicago U.S.A; d State Key Laboratory of Biotherapy, West China Hospital, Sichuan University, Chengdu, China

**Keywords:** Antimicrobial small molecule, endodontic diseases, *enterococcus faecalis*, ii-6s, microbial biofilm, root canal disinfection

## Abstract

**Background:**

Limitations of current intracanal irrigants such as sodium hypochlorite (NaOCl) and chlorhexidine (CHX) necessitate the development of novel antimicrobial agents to control endodontic infection.

**Aim:**

This study investigated the antimicrobial activities of a small molecule II-6s against *Enterococcus faecalis* associated with endodontic diseases.

**Methods:**

The susceptibility of *E. faecalis* to II-6s was evaluated by the microdilution method and time-kill assay. Microbial resistance was assessed by repeated exposure of *E. faecalis* to II-6s. Cytotoxicity of II-6s was evaluated by CCK-8 assay. Virulence gene expression of the II-6s-treated *E. faecalis* cells was measured by RT-qPCR. Bacterial reductions in the dentinal tubules were further assessed by confocal laser scanning microscopy.

**Results:**

II-6s exhibited potent antimicrobial activity against *E. faecalis* and down-regulated virulence-associated genes in *E. faecalis*. II-6s induced no drug resistance in *E. faecalis* with lower cytotoxicity as compared to NaOCl and CHX. More importantly, 0.003125% II-6s exhibited significant bactericidal effect against *E. faecalis* residing in the dentinal tubules, which was comparable to 5.25% NaOCl and 2% CHX.

**Conclusions:**

II-6s has excellent antimicrobial activity, moderate cytotoxicity and induces no drug resistance, and thus is a promising agent for the treatment of endodontic infection.

## Introduction

Endodontic diseases are results from inflammation and destruction of pulp and periradicular tissues, primarily initiated by oral biofilms and associated with multiple risk factors [1]. Root canal disinfection, aiming to disrupt biofilms and kill bacteria inside, is critical for successful endodontic treatment [2]. *Enterococcus faecalis* is the main species commonly isolated from root canals with persistent endodontic infection or post-treatment endodontic diseases [3,4]. Certain virulence factors of *E. faecalis*, such as collagen-binding protein, gelatinase, enterococcal surface protein and aggregation substance mediate the adherence and biofilm formation on the dentin surface of the root canal system [4–6]. *E. faecalis* can invade into dentinal tubules, compete with other microorganisms, and survive in the root canals with poor nutrition [3].

The success of endodontic treatment depends on effective disinfection of the root canal system [7]. Due to the complexity of the root canal systems, maximal eradication of microorganisms may be achieved by mechanical instrumentation supplemented with irrigation and intracanal medicaments [8]. Sodium hypochlorite (NaOCl) is one of the most commonly used irrigants during root canal treatment. It can dissolve necrotic tissue remnants as well as microbial biofilms [9]. The anti-biofilm efficacy of NaOCl improves with increasing concentrations [9,10]. However, the cytotoxicity of NaOCl on human cells is also positively correlated with its concentrations [11,12]. Chlorhexidine (CHX) is another commonly used endodontic irrigant. 2% CHX has decent antimicrobial activity against *E. faecalis* in the root canal [13]. However, repeated exposure to CHX could induce drug resistance in *E. faecalis* [14]. In addition, CHX is less effective against bacteria deep inside the biofilms due to the possible interaction of these cationic molecules with the matrix components of biofilms [15], further limiting its use in the control of endodontic disinfection. Therefore, alternative antimicrobial agents with comparable efficacy to NaOCl or CHX but fewer side effects are needed to better control *E. faecalis.*

**Figure 1.**
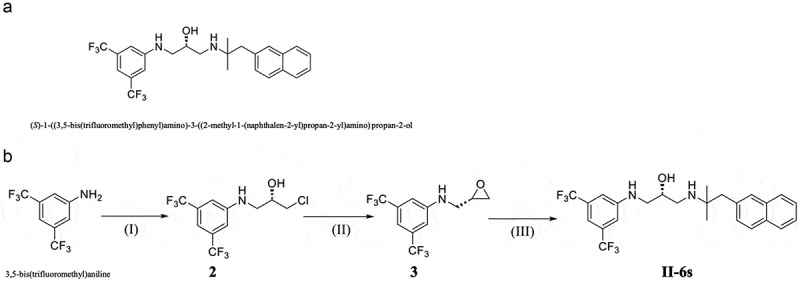
(**A**) Chemical structure of II-6s. (B) Synthesis of compound II-6s. Reagents and conditions are as follows: (**I**) (*R*)-epichlorhydrin, AcOH, 75°C, 8 h; (**II**) KF, MeCN, reflux, 5 h; (**III**) 2-methyl-1-(naphthalen-2-yl)propan-2-amine hydrochloride, Et_3_N, EtOH, reflux, 7 h

Recently, small molecules are promising for the control of biofilms due to excellent antimicrobial activity, low toxicity, and structural versatility [16]. Series of novel small molecules exhibit antimicrobial activities against both Gram-positive and Gram-negative bacteria [17,18]. Scientific screening and reasonable structural design are important for the development of novel antimicrobial small molecules. In the previous study, we performed a phenotypic screening of a small molecule library against *E. faecalis* and identified 23 compounds. We found that (*S*)-1-((3,5-bis(trifluoromethyl) phenyl)amino)-3-((2-methyl-1-(naphthalen-2-yl)propan-2-yl)amino)propan-2-ol, namely II-6s, exhibited antimicrobial activity against *E. faecalis* with a minimal inhibitory concentration (MIC) less than 16 μg/mL (Figure 1, Table S1). Our previous studies also reported that II-6s exhibited decent antimicrobial activity against methicillin-resistant *Staphylococcus aureus* and *Streptococcus mutans* with MIC of 2 μg/mL and 3.91 μg/mL, respectively [19,20]. All these data suggest that II-6s may have a good potential in the control of endodontic biofilms. Hence, in the present study, we comprehensively investigated the antimicrobial activity of II-6s against *E. faecalis in vitro* and in dentinal tubule models, aiming to explore its potential to be used in endodontic treatment.

## Materials and methods

### *Synthesis and preparation of* II-6s

II-6s was synthesized as described in our previous study [19]. Briefly, the synthesis of the target compound II-6s was accomplished in three steps: (I) A mixture of 3,5-bis(trifluoromethyl)aniline (1.31 mmol) and (*R*)-epichlorhydrin (6.55 mmol) in acetic acid (10 mL) was heated to 75°C. After 2 h, the reaction mixture was cooled to room temperature. The resulting mixture was concentrated under vacuum and the product was extracted three times with ethyl acetate (AcOEt, 100 mL for each time). The AcOEt extract was dried (Na_2_SO_4_), filtered and evaporated *in vacuo*. The crude was purified on the silica gel to yield *(R)*-1-((3,5-bis(trifluoromethyl)phenyl)amido)-3-chloropropan-2-ol (intermediate 2); (II) The solution of intermediate 2 (0.83 mmol) and KF (2.49 mmol) in acetonitrile (10 mL) was refluxed for 3 h. Then, the resulting mixture was filtered, and the filtrate was concentrated under vacuum, the product was purified on the silica gel to afford *(S)*-N-(oxiran-2-ylmethyl)-3,5-bis(trifluoromethyl)aniline (intermediate 3); (III) A mixture of intermediate 3 (0.29 mmol), 2-methyl-1-(naphthalen-2-yl)propan-2-amine hydrochloride (0.43 mmol), and triethylamine (0.43 mmol) in 15 mL of ethanol was refluxed for 7 h (TLC-monitoring). After cooling, the solvent was removed *in vacuo*. The crude product was purified on a silica gel column to give pure target compounds II-6s (Figure 1B). II-6s was prepared in DMSO at a stocking concentration of 100 mg/mL.

## Test bacteria and growth medium

*E. faecalis* ATCC4083 was kindly provided by the State Key Laboratory of Oral Diseases (Sichuan University, Chengdu, China). *E. faecalis* was routinely grown in brain heart infusion broth (BHI; Difco, Sparks, MD) at 37°C under anaerobic condition. Inoculum for the experiment was adjusted to 2 × 10^8^ CFU/ mL for *E. faecalis* based on the OD_600nm_ versus CFU/mL graph of bacteria and further 1:100 diluted in the growth culture. Medium was supplemented with 1% sucrose (designated BHIS) in the biofilm susceptibility test.

## Bacterial susceptibility test

The minimum inhibitory concentration (MIC) and minimum bactericidal concentration (MBC) of II-6s against *E. faecalis* were determined by the microdilution method in BHI, as described previously [21]. The concentrations of II-6s ranged from 0.98 to 1,000 μg/mL (two-fold dilutions). BHI broth containing equivalent DMSO (1% to 0.001%, *v/v*) was used as a solvent control for the possible growth inhibition of DMSO. CHX and NaOCl were used as positive controls, and bacteria grown without treatment were used as negative control and BHI broth as blank control.

## Biofilm susceptibility test

The minimum biofilm inhibitory concentration (MBIC) and minimum biofilm reduction concentration (MBRC) of II-6s against *E. faecalis* biofilm were determined in BHIS as described previously [18]. For the measurement of MBIC, *E. faecalis* was anaerobically grown in BHIS with twofold serial dilution of II-6s (0.12 to 125 μg/mL) in 96-well plates at 37°C for 24 h. A parallel study was also performed with BHIS as negative control and NaOCl/CHX as positive controls. After incubation, the supernatant from each well was decanted, and the adherent biofilm was washed three times with phosphate-buffered saline (PBS) to remove the planktonic cells. Fixed with methanol for 15 min, air dried at room temperature, and the biofilm was stained with 0.1% (wt/vol) crystal violet (Sigma) for 5 min. Rinsed with deionized water until the blank control wells were colorless and added 200 μL of 95% ethanol to each crystal violet-stained well. Subsequently, the plate was shaken 30 min at room temperature and the absorbance at 595 nm was recorded. For the measurement of MBRC, *E. faecalis* biofilm was performed in a 96-well plate containing BHIS by anaerobic incubation at 37°C for 24 h. The supernatant of preformed biofilm was decanted, and planktonic cells were removed by washing with sterile PBS. Fresh BHIS containing II-6s ranging from 0.12 to 125 μg/mL were added to the preformed biofilm and incubated at 37°C for 24 h. The negative control was preformed biofilms in BHIS without II-6s, and positive controls were biofilms treated with NaOCl/CHX. Similarly, the biofilm was fixed, air dried, stained and quantified as described above. The minimum biofilm inhibition concentration (MBIC_90_) was defined as the lowest agent concentration that showed 90% or more inhibition of biofilm formation. The minimum biofilm reduction concentration (MBRC_90_) was defined as the lowest agent concentration that showed 90% or more reduction of the pre-established biofilm.

## Time-kill assay

The kinetics killing effect of II-6s against *E. faecalis* was determined with the method modified from that reported by Koo et al. [22]. CHX and NaOCl were used as positive controls. The final concentrations of the test agents were at their MBC values, respectively. Tubes containing *E. faecalis* suspension (1 × 10^8^ CFU/mL) and antibacterial agents (MBC_II-6s_, 7.81 μg/mL; MBC_NaOCl_, 7,200 μg/mL; MBC_CHX_, 31.25 μg/mL) were incubated at 37°C. Samples were removed for counting colonies at 0, 2, 4, 6, 8 and 10 h, serially diluted with PBS, and spread onto BHI agar with the L-Spreader (Nest, China). The plates were incubated at 37°C for 24 h, and the number of colonies was counted. Negative control contained *E. faecalis* grown in BHI broth without II-6s, NaOCl or CHX. Killing curves were constructed by plotting the log_10_ CFU/mL versus time over 10 h. A bactericidal effect was defined as a more than 3-log_10_ CFU/mL decrease from the original inoculum.

## Bacterial drug resistance assays against antibacterial agents

Monitoring MICs of agent after serial passage of culture through sub-inhibitory concentrations has been proven to be an effective analysis of phenotypic adaptation [14]. CHX and NaOCl were used as positive controls. The MIC values of II-6s, NaOCl and CHX against *E. faecalis* were measured as described above. After determining the initial MICs, 20 μL of a bacterial suspension per well showing 1/2 MIC was mixed with 1,980 μL of BHI broth to eliminate the effect of drug carry-over. The resultant suspension was inoculated onto BHI agar and incubated at 37°C for 24 h. The overnight bacterial suspensions were then prepared for the next MIC test. The MIC determinations were repeatedly performed through 10 passages. Four times or higher increases in MIC over the initial MIC value indicated inducing bacterial resistance against each antibacterial agent [23].

## *In vitro* cytotoxicity/viability assay

The cytotoxicity of II-6s was performed on cells that would expose to an antimicrobial agent, with the aim of screening for concentrations that did not inhibit cell growth or induce cell death. Test cells were macrophages RAW264.7 (RAW264.7), human periodontal ligament fibroblasts (hPLFs) and human osteosarcoma MG63 cells (MG63). The concentrations used for each test agent referred to the determined MBRC to evaluate its cytotoxicity at the antimicrobial active dose. Cells were plated in 96-well plates at 10,000 cells/well with the Dulbecco’s modification of Eagle’s medium (DMEM) supplemented with 10% fetal bovine serum and 1% antibiotic-antimycotic. The cells were grown in a humidified environment with 5% CO_2_ at 37°C for 24 h. Then, treated with medium containing either II-6s (0.12 to 62.5 μg/mL) or positive control CHX (0.12 to 62.5 μg/mL) and NaOCl (225 to 14,400 μg/mL) for 5 min or 7 days. For the 7-day group, the medium containing test agents were refreshed every day. Then, cells were washed with PBS twice, and added the fresh medium (200 μL/well). The cytotoxicity of II-6s was determined by using the cell counting kit-8 (CCK-8, Dojindo, Kumamoto, Japan) assay. Each well was added with 10 μL of CCK-8, and after incubation in the CO_2_ incubator for 1 h to 1.5 h, absorbance was measured at the wavelength of 450 nm. The formula for the calculation of relative growth rate (RGR) is: RGR (%) = (A_450_ of test group − A_450_ of blank control)/(A_450_ of negative control − A_450_ of blank control) × 100%. Here, we employed dilution coefficient to evaluate the effective antimicrobial concentration and IC50 as reported by Barnhart et al. [12]. The dilution coefficient of II-6s was defined and calculated according to the following formula: dilution coefficient = MBRC/IC50 value. The lower dilution coefficient indicated the lower cytotoxicity.

## RNA isolation and quantitative reverse transcriptase PCR

Quantitative reverse transcriptase PCR (RT-qPCR) was used to determine the effects of II-6s on selected virulence factors at the transcriptional level. *E. faecalis* was grown in BHI medium until the cells reached the mid-logarithmic phase (2 × 10^8^ CFU/mL). The cells were collected by centrifugation and resuspended in BHI medium supplemented with sub-MIC levels of II-6s (1/2 MIC, 1.95 μg/mL; 1/4 MIC, 0.98 μg/mL; 1/8 MIC, 0.49 μg/mL) and incubated at 37°C for 2 h. Total bacterial RNA isolation was performed as described previously [24]. RNA purification and cDNA reverse transcription were performed with the PrimeScript^TM^ RT Reagent Kit (RR047A; Takara Bio, Japan). Real-time polymerase-chain reaction was performed with the LightCycler 480 II (Roche, Switzerland). Primers of the virulence-associated genes in *E. faecalis* were selected as previously described by Shepard et al. [25] (Table 1). 16S rRNA was used as internal control to normalize RNA concentration [26]. The reactions were repeated three times for each gene. The reaction mixture (25 μL) contained 2 × SYBR® Premix Ex Taq II (TaKaRa, Japan), template cDNA, forward primers (10 μM) and reverse primers (10 μM). Thermal cycling conditions were designated as follows: the first stage was 95°C for 30 s, and the second stage was 95°C for 5 s, 60°C for 30 s; 50 cycles. Threshold cycle values (*C_T_*) were determined, and the data were analyzed by LightCycler® 480 software (version 1.5.1) according to the 2 ^– ΔΔCT^ method.

**Table 1.** Primers used for quantitative real-time PCR

Genes	Forward primer (5ʹ-3ʹ)	Reverse primer (5ʹ-3ʹ)
*16S rRNA*	CCGAGTGCTTGCACTCAATTGG	CTCTTATGCCATGCGGCATAAAC
*ace*	CGGCGACTCAACGTTTGAC	TCCAGCCAAATCGCCTACTT
*esp*	GGAACGCCTTGGTATGCTAAC	GCCACTTTATCAGCCTGAACC
*gelE*	CGGAACATACTGCCGGTTTAGA	TGGATTAGATGCACCCGAAAT
*efaA*	TGGGACAGACCCTCACGAATA	CGCCTGTTTCTAAGTTCAAGCC
*asa*	GATACAAAGCCAATGTCGTTCCT	TAAAGAGTCGCCACGTTTCACA

## Antimicrobial activity of II-6s against *E. faecalis* in dentinal tubules

Thirty caries-free single-rooted human teeth extracted for orthodontic reasons were used in this study. Collection of extracted human teeth was approved by the Ethics Committee of West China Hospital of Stomatology, Sichuan University (WCHSIRB-ST-2018-07). The dentine specimens were prepared as described by Ma et al. [27]. Briefly, a root dentin block with a length of 4 mm was horizontally separated from each tooth at 1 mm below the cementoenamel junction by a diamond-coated saw (Struers Minitom; Struers, Copenhagen, Denmark) under continuous water cooling. The root canals inside the blocks were prepared with a Gates Glidden drill #6 (Tulsa Dentsply, Tulsa, OK) at 300 rpm under water cooling. Each cylindrical dentin block was fractured by first making a thin groove in the middle of the cemental specimen by using the diamond-coated saw and then fracturing the specimen with a blade and a hammer into two semicylindrical halves. Sixty semicylindrical dentin halves were shaped to 4 mm × 4 mm × 2 mm in size and were removed the root surface cement with waterproof silicon carbide abrasive papers (1,200 grit; Struers). All specimens were rinsed in an ultrasonic cleaner (Yuejin, China) with 5.25% NaOCl and 17% ethylene diamine tetraacetic acid (EDTA, pH = 7.0) each for 4 min to remove the smear layer, followed by rinsing in sterile water for another 1 min to remove the remnants. Then, the prepared specimens were sterilized by ethylene oxide. Five randomly selected specimens were subjected to scanning electron microscopy to confirm the absence of a smear layer, five specimens were subjected to confocal laser scanning microscopy analysis as blank controls, and another five specimens were incubated in BHI broth at 37°C for 24 h to ensure the absence of bacterial contamination. The rest of the prepared dentin specimens were placed on the bottom of 1.5 mL microtubes (Axygen, CA) and sealed with flowable composite resin (SHOFU, Japan). *E. faecalis* suspension (2 × 10^7^ CFU/mL) in BHI broth was added to each tube and centrifuged in sequence at 1,400 g, 2,000 g, 3,600 g, and 5,600 g twice each for 5 min. After centrifugation, all tubes were added fresh *E. faecalis* suspension and incubated at 37°C under anaerobic conditions for 3 weeks to allow biofilm growth and maturation in the dentinal tubules. The BHI broth was refreshed every 2 days. After incubation, the specimens were taken out of the tubes, removed of the surrounding composite, rinsed in sterile water, and the cemental surface was sealed by nail varnish.

To ensure the presence of *E. faecalis* after inoculation, five randomly selected specimens were examined by scanning electron microscopy. These specimens were fixed with 2.5% glutaraldehyde overnight and prepared to observe the dentin canals in the cross-sectional and longitudinal section. The specimens were then dehydrated with graded series of ethanol (30%, 50%, 70%, 80%, 90%, 95% and 100% for 15 min each), coated with iridium, and observed by scanning electron microscopy (Zeiss Leo 435 VP: Leo Electron Microscopy Ltd Cooperation Zeiss Leica, Cambridge, England) at magnifications of 5,000 ×, 10,000 ×, and 20,000 ×, respectively.

The 40 specimens were randomly divided into eight groups: PBS, 1% NaOCl, 5.25% NaOCl, 2% CHX, 0.001563% II-6s, 0.003125% II-6s, 0.00625% II-6s and 0.0125% II-6s. A droplet of 50 μL of each test agent was placed on the dentin surface of the root canal side for 5 min. The specimens were then washed in sterile water for 1 min and vertically fractured through the root canal into two halves as described previously to expose a fresh surface of dentin canals longitudinally for confocal laser scanning microscopy examination [27]. The fractured dentin pieces were stained with fluorescent LIVE/DEAD BacLight Bacterial Viability stain (Molecular Probes, Invitrogen, CA) containing SYTO 9 and propidium iodide according to the manufacturer’s instructions. The stained dentin pieces were imaged with a confocal laser scanning microscope (FV1000, Olympus, Tokyo, Japan) equipped with a 20 × objective lens and an additional zoom of 2 × in accordance with a previous study [27]. The confocal laser scanning microscopic data were processed by Imaris 7.2 software (Bitplane, Zürich, Switzerland). The proportion of dead cells was quantified with Image J software (NIH Image, Bethesda, MD) and COMSTAT (http://www.comstat.dk).

## Statistical analyses

For *in vitro* studies, all experiments were performed in triplicate and reproduced at least three separate times. Statistical analysis was performed with SPSS software, version 16.0 (SPSS, Inc., Chicago, IL). One-way analysis of variance (ANOVA) and *post hoc* multiple comparisons test were used to compare differences. Differences were considered significant at a *P* value of < 0.05.

## Results

### II-6s exhibits potent antimicrobial activity against E. faecalis

II-6s inhibited the growth of *E. faecalis* planktonic cells with an MIC of 3.91 μg/mL and killed *E. faecalis* with an MBC of 7.81 μg/mL (Table 2). II-6s was effective in inhibiting the formation of *E. faecalis* biofilm (MBIC = 1.95 μg/mL) and disrupting the pre-established biofilm at an MBRC of 31.25 μg/mL (Table 2). As positive controls, CHX and NaOCl inhibited the growth of *E. faecalis* at MIC of 1.95 μg/mL and 3,600 μg/mL, respectively. The MBC of CHX and NaOCl against *E. faecalis* was 31.25 μg/mL and 7,200 μg/mL, respectively. At higher concentrations, CHX and NaOCl were also able to inhibit the formation of *E. faecalis* biofilm (MBIC of 7.81 μg/mL and 7,200 μg/mL, respectively) and disrupt the pre-established biofilm with an MBRC of 62.50 μg/mL and 14,400 μg/mL, respectively (Table 2).

**Table 2.** MICs, MBCs, MBICs and MBRCs of test agents against *E. faecalis* strains

*E. faecalis* (μg/mL)	Planktonic cells		Biofilm	
	MIC	MBC	MBIC	MBRC
II-6s	3.91	7.81	1.95	31.25
NaOCl	3,600	7,200	7,200	14,400
CHX	1.95	31.25	7.81	62.50

MIC, minimum inhibitory concentration; MBC, minimum bactericidal concentration; MBIC, minimum biofilm inhibition concentration; MBRC, minimum biofilm reduction concentration

**Figure 2.**
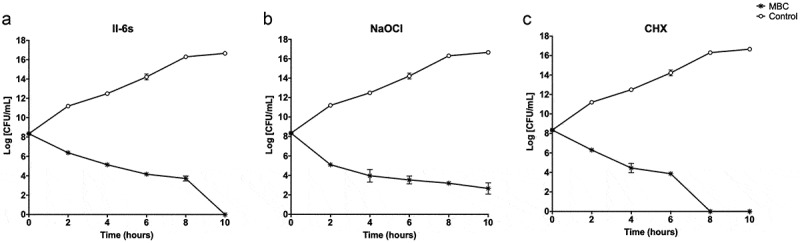
Kinetic killing effect of test agents on *E. faecalis* planktonic cultures. The concentrations of II-6s, NaOCl and CHX were at MBC values respectively. The untreated control was *E. faecalis* grown in BHI medium in the absence of II-6s, NaOCl or CHX. Data are means ± SD (n = 3)

The kinetic killing effects against *E. faecalis* were investigated by exposing the bacterial cells to MBC levels of testing agents. II-6s at 7.81 μg/mL showed bactericidal activity against *E. faecalis* after 4 h of incubation. This bactericidal effect was time dependent. After an exposure to 7.81 μg/mL of II-6s for 10 h, no viable *E. faecalis* was detected. The bactericidal activity of II-6s at 7.81 μg/mL was comparable to 31.25 μg/mL of chlorhexidine. However, although NaOCl at 7,200 μg/mL (0.72%) showed strong bactericidal activity after 2 h of incubation, approximately 1 × 10^3^ CFU/mL of *E. faecalis* was still detectable after 10 h of treatment (Figure 2).

## II-6s induced no drug resistance in *E. faecalis*

**Figure 3.**
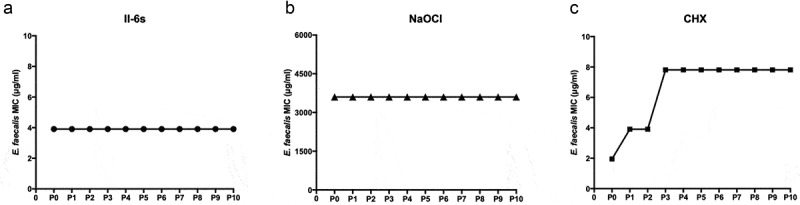
MIC values of test agents against *E. faecalis*. MICs were repeatedly measured from passage 0 (P0) to passage 10 (P10)

To evaluate whether II-6s induced bacterial resistance, the MICs of II-6s against *E. faecalis* at passages 0 to passages 10 are shown in Figure 3. CHX and NaOCl were used as positive controls. For II-6s, the MIC values of *E. faecalis* did not change from P0 to P10 (Figure 3A), which were constant at 3.91 μg/mL. For NaOCl, the MICs of *E. faecalis* were constant at 3,600 μg/mL from P0 to P10 (Figure 3B). However, the MICs of CHX against *E. faecalis* increased from 1.95 μg/mL to 3.91 μg/mL at P1, and then increased from 3.91 μg/mL to 7.81 μg/mL at P3 (Figure 3C), suggesting that *E. faecalis* developed antimicrobial resistance to CHX. In contrast, II-6s and NaOCl induced no drug resistance in *E. faecalis* after repeated exposures under our experimental conditions.

## II-6s shows lower cell cytotoxicity than NaOCl and CHX

**Figure 4.**
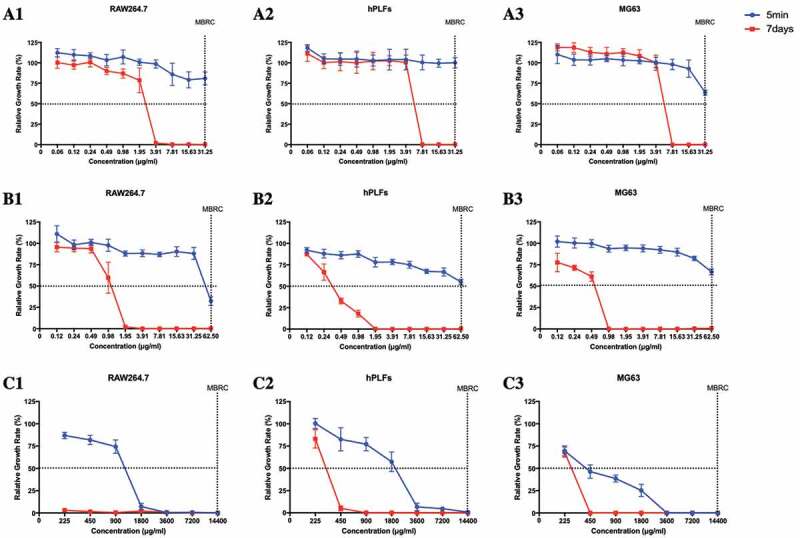
Cytotoxicity of test agents on macrophages 264.7, human periodontal ligament fibroblasts and human osteosarcoma MG63 cells. (**A1-A3**) Relative growth rate of RAW264.7, hPLFs and MG63 treated with II-6s; (**B1-B3**) Relative growth rate of RAW264.7, hPLFs and MG63 treated with CHX; (**C1-C3**) Relative growth rate of RAW264.7, hPLFs and MG63 treated with NaOCl. Data are means ± SD (n = 3). RAW264.7, macrophages RAW264.7; hPLFs, human periodontal ligament fibroblasts; MG63, human osteosarcoma MG63 cells

The cytotoxicity of II-6s against macrophages RAW264.7 (RAW264.7), human periodontal ligament fibroblasts (hPLFs) and human osteosarcoma MG63 cells (MG63) was evaluated by measuring the relative growth rate and dilution coefficient after drug exposure duration of 5 min and 7 days, respectively. Given the same exposure time, NaOCl and CHX significantly reduced the viability of the cultured cells as compared with II-6s. II-6s showed lower dilution coefficient than NaOCl and CHX, indicating a lower short-term (5 min) and long-term (7 days) cytotoxicity against test cells (Figure 4, Table 3).

**Table 3.** Dilution coefficient of test agents

Dilution coefficient	RAW264.7		hPLFs		MG63	
	5 min	7 days	5 min	7 days	5 min	7 days
II-6s	< 1	11.66	< 1	5.32	< 1	5.32
CHX	1.20	54.82	< 1	173.61	< 1	107.76
NaOCl	11.74	> 64	6.98	45.27	34.69	50.52

## II-6s down-regulates virulence-associated genes in *E. faecalis*

**Figure 5.**
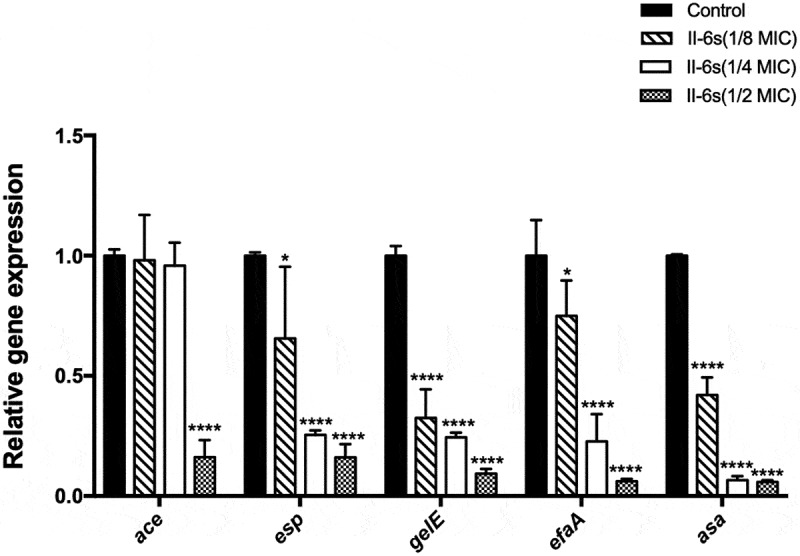
Effects of sub-MIC level of II-6s on the expression of virulence-associated genes in *E. faecalis*. Data are presented as means ±SD (n = 3). *, *P*< 0.05; ****, *P*< 0.0001 as compared to the untreated control group

RT-qPCR was used to evaluate the effect of sub-MIC levels of II-6s on virulence-associated genes in *E. faecalis* (Figure 5). The inhibitory effects of II-6s on the virulence-associated genes were dose-dependent. II-6s at 1.95 μg/mL (1/2 MIC) significantly suppressed the expression of *ace, esp, gelE, efaA* and *asa* by >75%, while II-6s at 0.98 μg/mL (1/4 MIC) and 0.49 μg/mL (1/8 MIC) also down-regulated the expression of *esp, gelE, efaA* and *asa* as compared with the untreated controls (*P* < 0.05).

## II-6s kills *E. faecalis* in dentinal tubules

**Figure 6.**
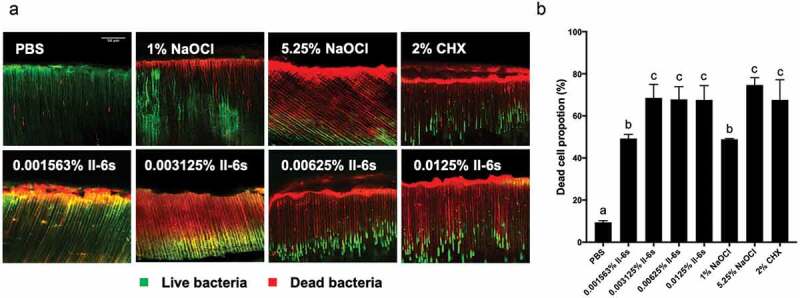
The antimicrobial effects of II-6s against 21-day-old *E. faecalis* biofilms in infected dentin tubules. (**A**) Representative image of dead/live bacteria within the dentinal tubules after treatment; live bacteria stained green; dead bacteria stained red; scale bar = 50 μm; (**B**) Proportion of dead bacteria after treatment; data are presented as mean ± SD (n = 5). Different lowercase letters indicate statistically significant differences between different treatment groups (*P* < 0.05)

The antimicrobial effects of II-6s were further evaluated against *E. faecalis* biofilms residing in the dentinal tubules. The presence of *E. faecalis* in the dentinal tubules was verified by scanning electron microscopy and confocal laser scanning microscopy (Figure S1, Figure S2). No visible microorganisms were observed inside the tubules before inoculation (Figure S1 A1-A4, Figure S2), and a large amount of *E. faecalis* invaded into the dentinal tubules after centrifugation and 21-day incubation (Figure S1 B1-B4, Figure 6A).

In the whole test area, 67% of bacteria were killed by 0.003125% II-6s, and 66% and 73% of bacteria were killed by 2% CHX and 5.25% NaOCl, respectively, after 5 min treatment. The proportion of the dead *E. faecalis* cells in the 1% NaOCl treated group, however, was only 48%. The antibacterial effect of II-6s at 0.003125% was comparable to that of 2% CHX and 5.25% NaOCl. Higher concentrations of II-6s (0.00625% and 0.0125%) killed 67% and 68% of *E. faecalis* in 5 min respectively, and no significant difference was observed as compared to II-6s at 0.003125% (Figure 6).

## Discussion

Endodontic biofilms play a critical role in the development and perseverance of endodontic diseases. The persistence of microbes within the root canal system is one of the major contributors to endodontic failure [28]. Among microorganisms detected in endodontically treated teeth with persistent infections, *E. faecalis* has a prevalence up to 77% [3]. *E. faecalis* can invade dentinal tubules, adhere to collagen, and form biofilms within the dentinal tubules [3]. It can also live within dentinal tubules and survive prolonged starvation [3]. Due to the anatomical complexity of the root canal system, unprepared canal surfaces still remain after mechanical instrumentation, regardless of instrumentation techniques [8]. Hence, irrigation is indispensable for the endodontic treatment. The application of antimicrobial irrigant supplements the elimination of microorganisms in the root canal systems [9]. NaOCl and CHX at higher concentrations exhibit strong antimicrobial activities against *E. faecalis*, but their cytotoxicity also increases with concentrations [11,12]. In addition, repeated use of antimicrobials such as CHX may induce drug resistance [29], further limiting its clinical applications. Therefore, it is necessary to develop alternative agents with supreme antimicrobial activity against persistent endodontic pathogens such as *E. faecalis* but with fewer side effects. Small molecules show good potential in controlling microbial biofilm due to their excellent stability, activity at low concentrations, and structural versatility [16]. This study identified a small molecule II-6s, which showed potent antimicrobial activity and moderate cytotoxicity relative to CHX and NaOCl, and induced no drug resistance in *E. faecalis*, representing a promising agent for the control of endodontic infection.

Elimination of *E. faecalis* residing in the dentinal tubules favors the clinical outcome of endodontic treatment [3,9]. Higher concentrations of NaOCl and CHX solutions exhibit antimicrobial activity against *E. faecalis* in the dentinal tubules [27]. In the current study, II-6s also showed remarkable bactericidal activity against *E. faecalis* in the dentinal tubules even at a very low concentration. Specifically, 0.003125% II-6s exhibited comparable antimicrobial effect to 5.25% NaOCl and 2% CHX, supporting its potential use as an alternative antimicrobial irrigant in the endodontic treatment. Since increase in propidium iodide-stained cells indicates membrane damage, the bactericidal effect of II-6s may be accredited to its ability to damage the cell membrane of *E. faecalis*. However, further study using flow cytometry is needed to substantiate this speculation.

Microbial adhesion and aggregation are critical for biofilm formation. Collagen-binding protein and gelatinase of *E. faecalis* mediate its binding to dentin [3]. The enterococcal surface protein, antigen A and aggregation substance of *E. faecalis* also contribute to its adhesion and biofilm formation [5,6,30]. Downregulation of these genes associated with biofilm formation in *E. faecalis* has proven effective in controlling biofilm [31]. Previous studies showed that mutations in *ace, gelE, asa, efaA* and *esp* resulted in fewer attached bacteria and less biofilm [31–33]. In the current study, we observed that a sub-MIC level of II-6s inhibited the biofilm formation of *E. faecalis*. We speculated that the underlying mechanism may involve the downregulation of *ace, esp, gelE, efaA* and *asa* that associated with the microbial adhesion and aggregation of *E. faecalis*.

Antimicrobial resistance has become a global problem that alarms the arbitrary use of broad-spectrum antimicrobials [14]. Repeated application of CHX could induce phenotypic adaptation in oral bacteria including *Streptococcus gordonii, E. faecalis, Fusobacterium nucleatum*, and *Porphyromonas gingivalis* [14]. Our study also found that repeated exposure to CHX could induce the adaption of *E. faecalis*, and consequently reduced the susceptibility of this bacterium to the CHX treatment. However, the susceptibility of *E. faecalis* to II-6s was consistent after repeated exposure, indicating the translational potential of II-6s as an endodontic irrigant to combat *E. faecalis*.

Biocompatibility is also an important clinical consideration of an endodontic irrigant within the effective doses. Especially in cases of open apex, root resorption, foramen enlargement and root perforation, the irrigant may extrude out of the canal and contact periapical tissues [34]. Human periodontal ligament fibroblasts, human osteosarcoma MG63 cells and macrophages are commonly used to evaluate the cytotoxicity of endodontic irrigants [35–37]. The current study evaluated both the short-term (5 min) and relatively long-term (7 days) cytotoxicity of II-6s on hPLFs, MG63, and macrophage RAW264.7. Short-term treatment was used to compare the instant cytotoxicity of II-6s, NaOCl and CHX considering the short exposure duration of oral cells to the endodontic irrigants. Long-term treatment was further used to evaluate the residual effect of irrigant on cell viability [35]. Lower cytotoxicity of II-6s against cultured cells was observed as compared to that of NaOCl and CHX. Of note, exposure of II-6s at its MBIC (1.95 μg/mL) for 7 days exhibited no significant inhibition on cell proliferation, suggesting that it may also be used as intracanal medicament at its low concentration. However, further studies are still needed to translate this promising small molecule compound into various applications in the endodontic infection control.

In conclusion, this study identified a small molecule II-6s and investigated its antimicrobial activity and cytotoxicity against *E. faecalis*. II-6s showed low cytotoxicity against human oral cells and exhibited potent antimicrobial activity against *E. faecalis* biofilms within dentinal tubules. In addition, repeated exposure to II-6s induced no drug resistance in *E. faecalis*. Taken together, this small molecule shows good potential as an alternative endodontic irrigant to supplement the control of endodontic infection.

## Supplementary Material

Supplemental Material
