# Supplementary material for: A small molecule II-6s inhibits Enterococcus faecalis biofilms
Source: J Oral Microbiol. 2021 Sep 14;13(1):1978756. doi: 10.1080/20002297.2021.1978756 (PMC8451641; doi:10.1080/20002297.2021.1978756)
Supplement: Supplemental Material [file ZJOM_A_1978756_SM1079.docx]

**Supplemental materials**

**Table S1.** Related to Figure 1. MICs of screened small molecules against *E. faecalis*

| *E. faecalis* | MIC (μg/mL) |
| --- | --- |
| I-1 | 62.50 |
| I-2 | 62.50 |
| I-3 | 62.50 |
| I-4 | 62.50 |
| I-5 | 62.50 |
| I-6 | 62.50 |
| I-7 | 62.50 |
| I-8 | 62.50 |
| I-9 | 62.50 |
| I-10 | 62.50 |
| I-11 | 62.50 |
| I-12 | 62.50 |
| I-13 | 31.25 |
| I-14 | 62.50 |
| I-15 | 62.50 |
| I-16 | 62.50 |
| II-2 | 31.25 |
| II-6s | 3.91 |
| NPSS | 31.25 |
| NPSR | 31.25 |
| KBR | 31.25 |
| KBS | 31.25 |
| KS | ＞ 62.50 |

**
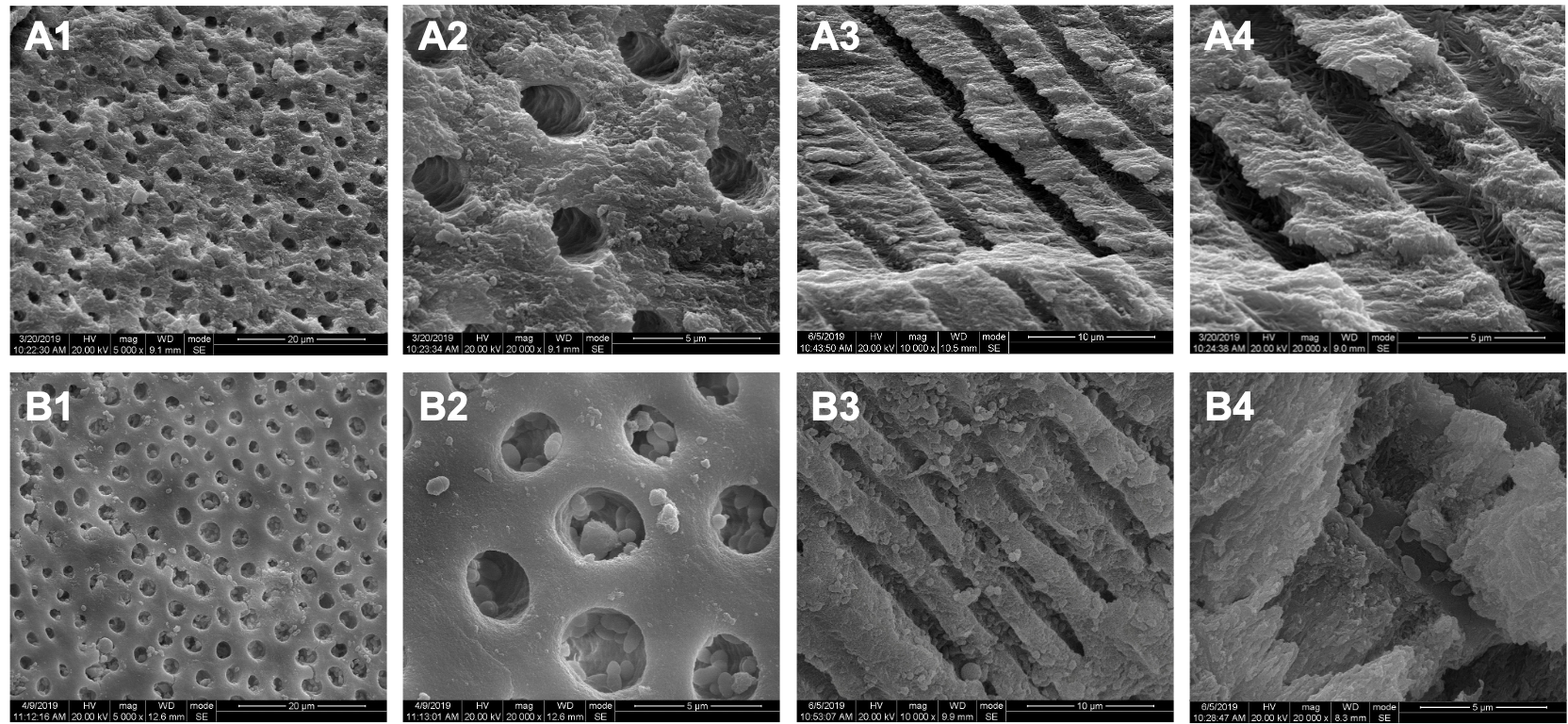
**

**Figure S1.** Related to Figure 6. Scanning electron microscopy of dentin surface before and after centrifugation of *E. faecalis*. (**A1– A4**) There were no residual microorganisms in the dentinal tubules before centrifugation; (**B1– B4**) Dentinal tubules were occupied by large amounts of *E. faecalis*. (**A1, B1**) × 5,000, (**A3, B3**) × 10,000, (**A2, B2, A4, B4**) ×20,000.

**
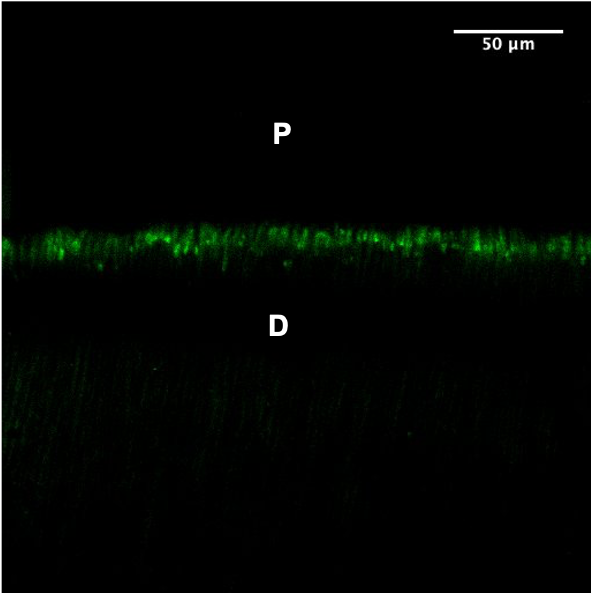
**

**Figure S2.** Related to Figure 6. Confocal laser scanning microscopy of unincubated dentin specimen. Only weak green fluorescence on the dentinal surface was observed. P, pulpal side; D, dentin; scale bar = 50 μm.
